# Use of intraoperative microvascular Doppler during subinguinal microsurgical varicocelectomy in children reduces complications

**DOI:** 10.55730/1300-0144.5849

**Published:** 2024-05-23

**Authors:** Cem KAYA, Sibel ERYILMAZ, Alparslan KAPISIZ, Ali ATAN, Ramazan KARABULUT, Zafer TÜRKYILMAZ, Kaan SÖNMEZ

**Affiliations:** 1 Department of Pediatric Surgery, Faculty of Medicine, Gazi University, Ankara, Turkiye; 2 Department of Urology, Faculty of Medicine, Gazi University, Ankara, Turkiye

**Keywords:** Varicocele, children, microvascular Doppler, microsurgery

## Abstract

**Background/Aim:**

This study assessed the impact of intraoperative microvascular Doppler ultrasonography (MDU) during microsurgical subinguinal varicocele correction in children.

**Materials and methods:**

Nineteen patients who underwent intraoperative MDU during subinguinal microsurgical varicocelectomy between 2021 and 2023 were included in this study. Each patient’s age, varicocele side, clinical examination findings, preoperative ultrasonography results, intraoperative findings, spermatic artery counts and findings in terms of MDU use, postoperative complications, and results were evaluated.

**Results:**

All varicoceles were on the left side and the average age of the patients was 15.2 years. The indications for varicocelectomy were testicular hypotrophy (n = 10) and scrotal pain or fullness (n = 9). When a surgical microscope was used, testicular artery pulsation was detected in only five patients, whereas it was detected in all cases when MDU was used. In 16 cases, a single testicular artery was identified, and two arteries were identified in three cases. Additionally, in a case where a spermatic vein was suspected, it was not ligated due to the detection of pulsation with an arterial pattern using MDU. Two to three lymphatic channels were isolated and preserved, an average of 7.5 vessels were ligated, and five external spermatic veins were identified and ligated. There were no complications, and six of the patients with testicular hypotrophy showed signs of the catch-up growth phenomenon.

**Conclusion:**

The use of MDU during subinguinal microsurgical varicocelectomy in children not only increases the success rate but also minimizes complications such as hydrocele and recurrence.

## 1. Introduction

Abnormal dilatation and tortuosity of the internal spermatic veins within the pampiniform plexus can result in a varicocele [1], which is the most prevalent condition that can be surgically corrected in teenagers. Varicoceles may cause scrotal pain or swelling. The main method used to diagnose varicoceles is a physical examination in a standing position [2]. High-grade varicoceles may result in the scrotum above the testicles having a “bag of worms” appearance. Varicoceles are categorized as grade I (palpable only during the Valsalva maneuver), grade II (palpable without the Valsalva maneuver), or grade III (visible without the need for palpation) based on physical examination results [3]. Varicoceles that are found during a physical examination are considered clinically important. Routine radiological imaging is not required to diagnose subclinical or clinical varicoceles clearly detected during a physical examination; however, scrotal Doppler ultrasonography (USG) is indicated in patients with questionable scrotal physical examination results. Testicular volume measurements, the presence of many dilated spermatic vessels, and the demonstration of reverse flow are indicators of a varicocele, as identified by color Doppler USG. The European Society of Urogenital Radiology Scrotal and Penile Imaging Working Group (ESUR-SPIWG) has stated that a varicocele can be diagnosed with a maximal diameter of ≥3 mm [4,5], and reflux longer than 2 s during the Valsalva maneuver while the patient is upright is considered abnormal [4,6].

As the indications for varicocelectomy in adolescents with varicoceles are not clear, it is challenging to apply a standard approach when treating these patients. Differences in the size of the right and left testis, scrotal pain, hormone levels, and, if possible, semen analysis parameters are considered important factors to take into account when deciding whether to perform a varicocelectomy [7]. A semen analysis should be performed for adult patients with a varicocele; however, it is not possible to perform a semen analysis for all pediatric patients with a varicocele because the first ejaculation usually occurs 1.5 years after the onset of puberty [8]. Hence, semen analysis may be performed for patients aged ≥17 years and it is not appropriate for younger patients due to ethical concerns [9]. Varicoceles in adolescents can be managed with radiological techniques, surgery, or observation with follow-up [10].

Despite some technical difficulties associated with the technique, the subinguinal approach has gained popularity and is now the primary approach used in varicocelectomies. It can be performed under magnification with loops (2.5× to 4.5×) or microscopes (8× to 15×). While this method is the gold standard for treating adults, there are limited data available on the successful use of this microsurgical subinguinal technique for treating adolescents [11]. Nevertheless, performing subinguinal microsurgical varicocelectomies in pediatric patients is increasing in popularity [11]. The key challenges associated with using this method in pediatric patients are the detection and preservation of the testicular artery.

In recent years, studies have shown that there are more positive results and fewer problems when intraoperative Doppler USG is used [12]. Currently, there is limited research available on the use of intraoperative microvascular Doppler ultrasonography (MDU) for subinguinal microsurgical varicocele repair in children, who have smaller vessel diameters and lower blood pressure than adults. Thus, we report on the use of intraoperative MDU for the identification and preservation of spermatic arteries during microsurgical subinguinal varicocelectomy in children.

## 2. Materials and methods

This study was planned after approval was obtained from the Gazi University Ethics Committee (04.09.2023-689). Nineteen patients who were referred to our clinic between January 2021 and July 2023 due to the presence of a clinically palpable varicocele were included in this study. Each patient’s age, varicocele-related symptoms, physical examination findings (e.g., varicocele side, testicular volume), and preoperative scrotal color Doppler USG results in the supine and standing positions were evaluated. Scrotal pain was evaluated using the patient’s/their family’s anamnesis; no pain scales were used to evaluate scrotal pain. Semen analysis was performed for two patients aged over 17 years after obtaining consent for sperm analysis from the patients and their families. The patients underwent subinguinal microsurgical varicocelectomy with intraoperative MDU (20 MHz vascular Doppler system, Vascular Technology Inc., Nashua, NH, USA). The intraoperative findings, postoperative complications, and outcomes were evaluated.

### 2.1. Subinguinal microsurgery and microvascular Doppler usage

Every operation was performed with general anesthesia. A 2–3 cm subinguinal incision was made 1 cm below the external inguinal ring. The spermatic cord and components were released upon reaching the outer ring. A Penrose drain was introduced via the incision and extracted. Any external spermatic veins found were tied up at the base of the groin using 4/0 silk sutures. After the exterior spermatic fascia was dissected, the internal spermatic vessels were examined, dissected, and ligated with the use of a surgical microscope (S88, Carl Zeiss Meditec, Jena, Germany) at 10× magnification. A Zeiss operating microscope was used for the dissection, which was performed at 8× to 15× magnification. Testicular arterial blood flow was precisely identified by meticulously dissecting the spermatic cord. A warm sterile saline solution was used to aid the identification of the visual arterial pulse. Minor bleeding was controlled using microsurgical bipolar coagulation. After all the arteries in the cord were identified and preserved, the internal spermatic veins were ligated with 4/0 silk sutures and separated. All the identified lymphatic structures were carefully preserved. These appeared as transparent tubular structures under the microscope, as shown in the video provided (Supplemental video). As experience with the use of a microscope increases, it becomes easier to distinguish vascular and lymphatic structures. For the intraoperative MDU, a 20-MHz MVD transceiver with a 0.5/1.5-mm-diameter probe was systematically used to scan all the spermatic vessels prior to ligation, irrespective of their visual appearance and pulsation. If a pulse (whistle) was heard when an artery was touched, it was identified and preserved.

The procedure was continued until pulsation of the testicular artery was obtained in all cases and the number of arteries at this level was recorded. Unintentional damage during the procedure, an inability to recognize an artery, any accidental arterial ligations, any injuries, and the duration of the surgery were documented (Supplemental video).

## 3. Results

The patients were aged between 11 and 18 years (mean age: 15.2 years). The varicocele was on the left side in all cases. After a physical examination, 13 patients were diagnosed with grade III varicocele and six patients were diagnosed with grade II varicocele. USG findings showed that in 10 patients, the testicular volume on the varicocele side was 20% or 2 mL less than the testicular volume on the opposite side.

The indications for varicocelectomy were testicular hypotrophy in 10 patients and scrotal discomfort or fullness in nine patients. Testicular hypotrophy was detected by the parents in three patients and during routine physical examination in seven patients. Parents stated that when they checked their child’s testicles due to complaints about their child’s testicles, they noticed that the left-side testicle was small.

During the surgical procedures, testicular artery pulsation was visible in only five patients when a surgical microscope was used to detect pulsation. However, arterial pulsation was detected in all cases when the MDU device was used. In addition, a single testicular artery was found in 16 patients, and two arteries were detected in three patients when the MDU device was used (Table). In one case, it was not possible to visually distinguish whether a vascular structure was an artery or a vein. In this case, the vascular structure was determined to be an artery using MDU.

In each case, two to three lymphatic channels were identified and maintained. In five patients, external spermatic veins were identified and ligated (26.3%). The number of internal spermatic veins that were ligated varied from six to nine (average: 7.5 vessels). The average skin-to-skin surgery time was 56 min (range: 40–70 min). The average postoperative follow-up period was 11.5 months (range: 2–30 months). No testicular atrophy, wound site infection, postoperative hydrocele, recurrence, or epididymitis was recorded during the follow-up period (Table). Over the 1-year follow-up period, six of the 10 patients who had testicular hypotrophy demonstrated catch-up growth.

## 4. Discussion

Microsurgical subinguinal varicocelectomy has become a popular alternative as magnification permits all dilated veins to be recognized and ligated and the arteries and lymphatics in the pampiniform plexus to be preserved. Hydrocele formation has been found to occur in 1%–24%, 3%–32%, 2%–12%, 0%–5%, and 0%–3% of cases treated with retroperitoneal, macroscopic inguinal, laparoscopic, radiological, and microsurgical techniques, respectively. Recurrence has been recorded in 0%–18%, 12%–15%, 1%–15%, 15%–25%, and 0%–3% of cases treated with retroperitoneal, macroscopic inguinal, laparoscopic, radiological, and microsurgical techniques, respectively. Reoperation has been required in 4%–8%, 3.4%, 2%–4%, 5%–9.9%, and 0%–0.01% of cases treated with retroperitoneal, macroscopic inguinal, laparoscopic, radiological, and microsurgical techniques, respectively [2].

Varicoceles occur in approximately 15% of males in general; however, the frequency can reach 35% in situations of primary infertility and 75% in situations of secondary infertility. Infertility, chronic discomfort, and testicular hypotrophy in children and teenagers are the major reasons for performing surgery to correct varicoceles. Laparoscopic repair, microsurgical repair via groin or subinguinal incision, and classic inguinal (Ivanissevich) or high retroperitoneal (Palomo) techniques are the surgical alternatives for varicocele correction [13,14]. There are benefits and drawbacks associated with each of these approaches and the results of several studies have been conflicting [15]. Repairing a varicocele might result in hydrocele creation, recurrence, a permanent varicocele, or, in rare cases, testicular atrophy.

Microsurgical varicocelectomy (MIV) is the most commonly used surgical method for treating varicoceles in adults because of its high success rate and low risk of complications. It is also frequently performed for pediatric patients [13,16]. In a study by Carbone and Merhoff, among 139 adult men who underwent microsurgical varicocele ligation, only one had varicocele recurrence (0.7%) and only four had complications (2.9%). Hydrocele formation, the most frequent complication following nonmicrosurgical varicocele ligation (reported rates: 5.4%–7.2%), did not occur in any of the patients [17]. It has also been reported that hydroceles can develop even when the 10% Palomo method is used [18,19].

Without magnification, the risk of recurrence after inguinal varicocelectomy is 15%–16%, and the inadvertent closure of the testicular lymphatic vessels during surgery increases the risk of postoperative hydrocele by 10% [20]. Similarly, in laparoscopic varicocele repair, recurrence rates of 2.2%–25% have been reported, as well as a hydrocele formation rate of 12.5% [18,21].

Performing MIV in children is emerging as a current trend, and Yaman et al. successfully employed this method in adolescents and children. In their study that included 92 children, no hydrocele formation was observed, while one case of recurrence was reported [18]. In contrast, Schiff et al. used a subinguinal microsurgical approach and reported no recurrence and one persistent hydrocele among 74 children [22]. Additionally, Choi et al. reported a recurrence rate of 20% in 25 patients when a microsurgical approach was utilized to ligate gubernacular veins and a rate of only 6.1% (2 out of 33 patients) when the same approach was utilized without inducing testicular torsion. They also reported that no hydrocele formation or atrophy was observed in any of the 58 patients following varicocele repair [13]. Although our study included only a limited number of patients (n = 19) who underwent subinguinal microsurgical varicocelectomy with the use of MDU, no recurrence, hydrocele formation, or testicular atrophy were observed.

Multiple branches of the testicular artery are detected in the subinguinal region in up to 40% of cases during MSV [23–26]. Jarow et al. [25] reported finding multiple testicular artery branches in 81% of their histological analyses. Given the reported presence of these branches, it is believed that the unintentional ligation of tiny (secondary) internal testicular artery branches occurs more often than is officially recorded. Cocuzza et al. [26] reported that the incidence of unintentional arterial ligation was 1.1% in adult patients in the absence of Doppler guidance. Since these ligations may negatively impact spermatogenic recovery or an increase in fertility, every attempt should be made to avoid inadvertent arterial ligations during varicocelectomy.

Factors such as small vessel size, aggressive manipulation of vessels during dissection, difficulty in identifying arterial pulsations leading to spasms, and vessels being buried near or beneath complex vessel branches could contribute to arterial injury [27]. According to Grober et al. [24], improvement in semen characteristics does not correlate with the number of preserved arteries following varicocele surgery. As such, the preservation of a single testicular artery does not guarantee the maintenance or even the optimization of testicular function. In addition, according to available data, there appears to be no correlation between preoperative characteristics and the number of testicular arteries found following surgery.

Thus, to guarantee the greatest possible preservation of arterial blood flow to the testicles, the most recent technologies should be utilized in varicocele surgery, such as optical magnification, advanced microsurgical tools and techniques, and vascular Doppler USG. Additionally, it has been demonstrated that microsurgical varicocelectomy produces better results in terms of the surviving vessel stumps and the overall motility and concentration of sperm [28]. In the study by Cocuzza et al., which was conducted with adult patients, it was also emphasized that MDU guidance allowed for the ligation of more spermatogenic vessels [26].

Although our study had a relatively short follow-up period, growth was detected in 60% (6 out of 10) of patients with testicular hypotrophy. Sinanoglu et al. stated that catch-up growth started from the ninth postoperative month and continued until the 36th postoperative month [29]. In another study, testicular growth began within 12 months after varicocelectomy in adolescents aged between 15 and 19 years [30]. Catch-up growth rates of 73%–90% have been reported in some series [31,32].

Due to the limited use and reliability of sperm analysis in children and adolescents for varicocele follow-up and treatment and considering the occurrence of catch-up growth following testicular repair, we recommend that all the most recently developed and available equipment be used for treating varicoceles.

The limitations of this study include its nonprospective design, its small patient sample size, and the lack of consolidation of the results due to the surgeries being performed by multiple surgeons. Therefore, this study should be supported in the future, either with more data from the same center or with more patients from multiple centers. Despite these limitations, this pioneering study on the use of MDU during subinguinal microsurgical varicocelectomy has achieved successful outcomes.

## 5. Conclusion

Children undergoing subinguinal microsurgical varicocelectomy benefit from the use of MDU; it has been shown to reduce the risk of complications, including hydrocele formation and varicocele recurrence. However, further research is needed to explore the use of MDU in both adults and children undergoing subinguinal microsurgical varicocelectomy and to determine its benefits and drawbacks and how it compares to other methods.

## Supplementary Information



## Figures and Tables

**Table:** Characteristics of 19 pediatric patients with left varicocele.

Grade of varicocele (grade/patients)	III/13 II/6
Testicular hypotrophy	10
Scrotal discomfort or fullness	9
Number of spermatic arteries (n/patients)	1/16 2/3
Number of ligated spermatic veins (number (range))	7.5 (6–9)
Number of external spermatic veins	5 (26.3%)
Recurrence/hydrocele/testicular atrophy	0
Catch-up growth phenomenon (n/%)	6/60%
Mean follow-up, months (range)	11.5 (2–30)
